# Effect of Hormonal Treatments on Cannabinoid Content Levels in Female Hemp (*Cannabis sativa* L.) Inflorescences

**DOI:** 10.3390/ijms26073445

**Published:** 2025-04-07

**Authors:** Juyoung Kim, Dong-Gun Kim, Tae Hyun Ha, Woon Ji Kim, Jaihyunk Ryu, Jin-Baek Kim, Sang Hoon Kim

**Affiliations:** Advanced Radiation Technology Institute, Korea Atomic Energy Research Institute, Jeongeup 56212, Republic of Korea; jykim83@kaeri.re.kr (J.K.); dgkim-@kakao.com (D.-G.K.); hath1002@kaeri.re.kr (T.H.H.); wjkim0101@kaeri.re.kr (W.J.K.); jhryu@kaeri.re.kr (J.R.); jbkim74@kaeri.re.kr (J.-B.K.)

**Keywords:** *Cannabis sativa*, γ-aminobutyric acid (GABA), abscisic acid (ABA), salicylic acid (SA), cannabinoids

## Abstract

The diverse hormonal treatments applied to hemp (*Cannabis sativa* L.) carry significant implications for cultivation, and yield optimization across a range of applications, including fiber, seed, oil production, and the enhancement of medicinal compounds. However, there is no evidence concerning the long-term consequences of hormonal treatment. To determine the connection between the effects of hormonal treatment and cannabinoid accumulation, hemp plants were treated with γ-aminobutyric acid (GABA), abscisic acid (ABA), and salicylic acid (SA) to investigate their effects on gene expression and cannabinoid content levels in female inflorescences at 3 days and 4 weeks after treatment. The treatments influenced the transcript levels of five key cannabinoid biosynthesis genes, with 1.0 mM GABA significantly increasing *OAC*, *THCAS*, and *CBCAS* transcripts within 48 to 72 h. Additionally, 1.0 mM GABA led to a significant increase in tetrahydrocannabinol content by day three and significant increases in total cannabidiol and cannabinoid by week four. In addition, both ABA and SA induced transient, dose-dependent increases or decreases in gene expressions, but cannabinoid accumulation at 4 weeks showed no significant changes compared to the control. These results provide valuable insights for hormonal application in cultivation and the development of traits that enhance cannabinoid production in cannabis cultivation, which could significantly contribute to optimizing industrial applications.

## 1. Introduction

Medicinal plants are the most important source of commercially produced secondary metabolites. Among these, *Cannabis sativa* L. is an annual dioecious plant that primarily produces secondary metabolites known as cannabinoids [1]. Hemp [cannabis, Cannabaceae, total tetrahydrocannabinol (THC) < 0.3% *w*/*w*], classified as an industrial-purpose cultivar, is a herbaceous flowering plant that is emerging as a component for future industries to develop fiber- or plant-based medicines [2]. Cannabis’s major medicinal products are known as THC and cannabidiol (CBD), which are the phytocannabinoids of more than 100 identified cannabinoids in cannabis plants [3]. They have a range of pharmacological properties, as they can treat chronic pain, relieve seizures, and reduce muscle spasms [4,5]. The majority of cannabinoid production occurs in unfertilized female flowers, especially in glandular trichomes on the female flower surface [1]. Therefore, enhancing cannabinoid accumulation in female flowers has been a critical goal of industrial application.

Various strategies are used to enhance the production of secondary metabolites, with hormone treatments, modulation of membrane permeability, and in situ product removal being among the most prevalent. Membrane permeability can be altered through the application of chemical or physical treatments [6]. A wide variety of methods and agents have been used to increase membrane permeability, including chemical treatments (e.g., solutions of high ionic strength, external pH change, dimethylsulfoxide, Tween 20 (polyoxyethylene sorbitan monolaurate) and chitosan addition) and physical treatments (e.g., pulsed electric fields, ultrasound, and high hydrostatic pressure) [6]. In the case of hormone treatments, readily available and inexpensive compounds, such as γ-aminobutyric acid (GABA), abscisic acid (ABA), and salicylic acid (SA), have been identified as inducers that can alter the bioactivity of plant secondary metabolites and related functionalities. Previous studies have shown that GABA, its plant hormone-like activity, plays a role in pH regulation, nitrogen storage, development, stress responses [7,8], and plant reproduction [9]. The in planta concentration of GABA is usually low under normal conditions but increases rapidly in response to biotic and abiotic stresses [10]. In addition, GABA contributes to the regulation of gene expression in various plants under stress conditions [11]. Meanwhile, ABA regulates many aspects of plant growth and development [12], and SA is a general signaling molecule responsible for improving tolerance to some biotic and abiotic stresses. It also stimulates the biosynthesis of specific secondary metabolites in various plants [13,14,15,16]. In addition, SA has been demonstrated to increase the glandular trichome size and density in several species [17,18]. These findings provide strong evidence that SA can positively regulate cannabinoid biosynthesis. However, the above studies on the exogenous application of hormones typically examined the expression and cannabinoid content at 3 days but not at more extended periods after hormone treatment on the cannabis plants’ flowers. Therefore, studies on the effects of different hormones or chemicals on the accumulation of cannabinoids and on the transcript levels of the genes involved in their biosynthesis may provide information about how to increase cannabinoid content and which genes are involved in this process. The genes responding to external hormone treatment, which are important for cannabinoid accumulation, can then be targeted in breeding programs to produce new cultivars with desirable traits.

The types and contents of cannabinoids, which are determined by genes encoding enzymes in the cannabinoid biosynthetic pathway (and by factors that influence the expression of those genes), affect the plant’s medicinal value [3] (Supplementary Figure S1). The precursors for cannabinoid biosynthesis are derived from two pathways: the hexanoate and the plastidial 2-C-methyl-D-erythritol 4-phosphate (MEP) pathways [19]. In the former pathway, fatty acid degradation activates a pathway in which acyl-activating enzyme produces hexanoyl-CoA, which is a substrate for polyketide synthase (olivetol synthase). Olivetols are then transformed by olivetolic acid cyclase (OAC) into olivetolic acid (OLA). The MEP pathway produces geranyl pyrophosphate (GPP) through sequential reactions. Then, the two substrates (OLA and GPP) produced in the two pathways are converted into cannabigerolic acid (CBGA) by an aromatic prenyltransferase [PT or cannabigerolic acid synthase (CBGAS)]. CBGA serves as the substrate for three oxydocyclases: cannabidiolic acid synthase (CBDAS), ∆9-tetrahydrocannabinolic acid synthase (THCAS), and cannabichromenic acid synthase (CBCAS) [19,20,21,22]. After the decarboxylation process, three neutral cannabinoids (tetrahydrocannabinol, THC; cannabidiol, CBD; and cannabichromene, CBC) are derived from acidic cannabinoid forms [1,23]. CBGAS is a prenyltransferase, whereas THCAS, CBDAS, and CBCAS are all closely related oxidocyclases [24]. Almost all cannabis plants contain an active CBGAS that converts precursor molecules into CBGA, which is then metabolized to form THCA or CBDA. CBCA is similarly synthesized from CBGA by CBCAS. However, this is less abundant, and its effects on cannabinoid accumulation are not as well researched. Through these pathways, five enzymes (OAC, PT, THCAS, CBDAS, and CBCAS) play a significant role in producing cannabinoids (cannabigerol (CBG), THC, CBD, and CBC).

The current regulatory environment surrounding hemp is complex and rapidly evolving. In legal terms, hemp plants are generally considered to have concentrations of the restricted psychoactive compound THC below a certain legal threshold that varies depending on the country, but it is typically 0.3% (*w*/*w*) by dry weight in international regulatory frameworks [25]. The presence or absence of THCAS and CBDAS differs among cannabis cultivars and is the most important factor in terms of a plant’s phytocannabinoid profile (e.g., whether there is high or low THC content in the female inflorescences). Plants that produce high levels of THCA (up to 20% of the dried flower mass) are known as marijuana and have strong THCAS and low CBDAS activity. Plants with very low THCA and moderate CBDA levels, known as hemp, have predominantly CBDAS activity. Plants with both CBDAS and THCAS activity produce a mixture of THCA and CBDA [26,27]. In this regard, exploring the effects of hormone treatments on the THC content of hemp plants in relation to international standards is an important pursuit.

Understanding the effects of specific hormone treatments on plant tissues and various metabolic pathways is fundamental for designing protocols that enhance the production of secondary metabolites. Appropriate standardized protocols for spraying plants to induce the production of specific metabolites, such as THC and CBD, which are mainly produced in female inflorescences, offer many advantages over other induction methods. Therefore, in the present study, we determined the effects of spraying cannabis plants with GABA, ABA, and SA on the content levels of THC and CBD in female inflorescence. We also determined how treatments with different concentrations of GABA, ABA, and SA affected the expression of five key genes involved in THC and CBD biosynthesis, and the relationships between gene transcript levels and cannabinoid content. These results provide fundamental insights into the biology of cannabis, identify the genes important for cannabinoid accumulation, and reveal which hormone treatments can maximize the production of important bioactive compounds.

## 2. Results

### 2.1. Differential Expression of Cannabinoid Biosynthesis Genes After Hormone Treatments

**Figure 1:**
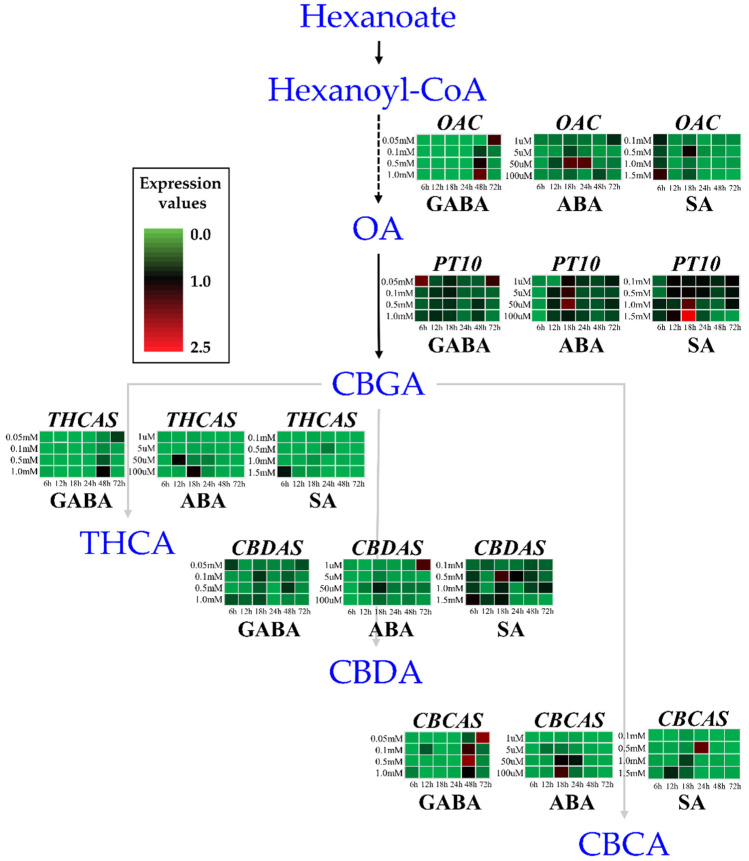
Transcript levels of five major structural genes in the cannabinoid biosynthetic pathway. For each gene, transcript levels at different times after treatment with hormones at a range of concentrations are displayed as a heatmap based on normalized Ct values. Each value for color scale was derived from 2^−∆∆Ct^/the largest 2^−∆∆Ct^ and multiplying by 2.5 for range 0–2.5. Statistical significance by Duncan’s multiple range test (*p* < 0.05, *n* = 3) is listed in Supplementary Table S3. GABA, γ-aminobutyric acid; ABA, abscisic acid; SA, salicylic acid; *OAC*, encodes olivetolic acid cyclase; *PT10*, prenyltransferase 10; *THCAS*, tetrahydrocannabinolic acid synthase; *CBDAS*, cannabidiolic acid synthase; *CBCAS*, cannabichromenic acid synthase.

To identify the relationship between hormones and gene expression involved in cannabinoid biosynthesis, the transcript profiles of five key genes of cannabis (*OAC*, *PT10*, *THCAS*, *CBDAS*, and *CBCAS*) were analyzed using qRT-PCR. These analyses were conducted at 6, 12, 18, 24, 48, and 72 h after treatment with the three hormones (GABA, ABA, and SA) at various concentrations, as described in the Materials and Methods section. After treatment with GABA, the relative expression (against a 0 mM control) of *OAC*, *THCAS*, and *CBCAS* showed a pattern of increased transcript levels at 48 and 72 h, whereas their transcripts were absent or showed decreased levels at 6, 12, 18, and 24 h (Figure 1). These results confirmed the dose-dependent manner in which GABA induces the cannabinoid biosynthesis pathway, with a critical induction point of 0.5 mM or 1.0 mM at 48 h (Figure 1). In contrast to the expression pattern of *OAC*, *THCAS*, and *CBCAS*, the relative expression of *PT10* and *CBDAS* after GABA treatment exhibited an atypical pattern.

Treatment with ABA affected the expression of all five genes, with a pattern of increased transcript levels of the five genes beyond 12 h after all concentrations of ABA treatments (Figure 1). In particular, *OAC*, *PT10*, and *CBDAS* expressions were higher in 50 µM of ABA at 18 h, and *THCAS* and *CBCAS* expressions were higher in 100 µM at 18 h. Exceptionally, the highest transcript level of *CBDAS* was at 72 h after treatment with 1 µM ABA (Figure 1).

A typical expression pattern was not detected in the SA treatment. However, the highest transcript levels of *OAC*, *PT10*, and *THCAS* were in the 1.5 mM SA treatment, with peak transcript levels of *OAC* and *THCAS* at 6 h post-treatment and a peak transcript level of *PT10* at 18 h. In contrast, *CBDAS* and *CBCAS* expressions after SA treatment were higher in the 0.5 mM SA at 18 h and 24 h, respectively (Figure 1). The results of SA treatment confirmed that the expression patterns of the cannabinoid biosynthetic pathway genes were not time- or dose-dependent.

### 2.2. Effect of Three Hormones (GABA, ABA, and SA) on Cannabinoid Contents

**Figure 2:**
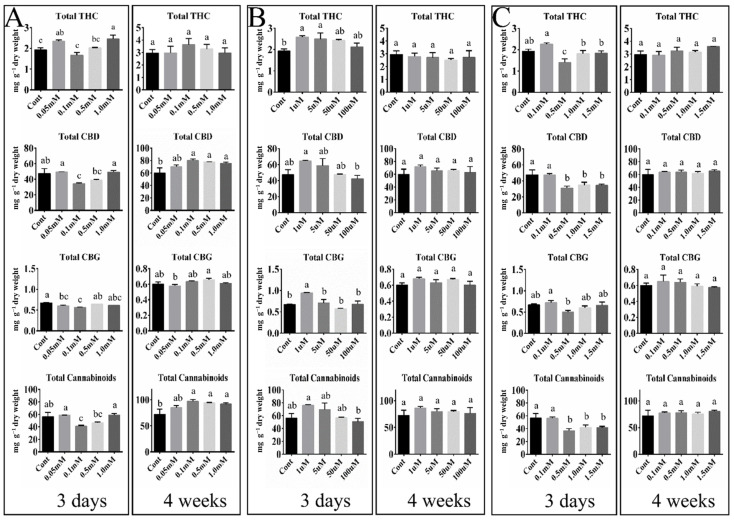
Contents of major cannabinoids in hemp inflorescences at 3 days and 4 weeks after hormone treatments. (**A**) GABA treatment; (**B**) ABA treatment; (**C**) SA treatment. THC, tetrahydrocannabinol; CBD, cannabidiol; CBG, cannabigerol. Mean values with the same letter are not significantly different at the 5% probability level (Duncan’s multiple range test, *n* = 3). See Table S1 for details.

To investigate the cannabinoid content of female hemp inflorescences, the contents of four major cannabinoid classes (total THC, CBD, CBG, and cannabinoids) were measured using high-performance liquid chromatography (HPLC) analysis (Supplementary Table S1). These analyses were conducted for inflorescences collected at 3 days and 4 weeks after treatment with GABA, ABA, or SA because the accumulation of cannabinoids normally peaks at 6–8 weeks after the short day transition [28], which means a somewhat long duration may be required for the highest accumulation after hormone treatment (Figure 2).

At 3 days post-treatment with GABA, significant increases in total THC content were observed in the 0.05 mM and 1.0 mM GABA-treated groups compared to the control (*p* < 0.05) whereas other cannabinoid classes (total CBD, CBG, and cannabinoids) showed content levels similar or lower to the control (Figure 2A). By week four, no significant differences in THC content levels were noted between the GABA-treated samples and the control at any concentration. Nevertheless, after 4 weeks, a significant increase in the classes of total CBD and cannabinoids was observed compared to the control (Figure 2A). In addition, total cannabinoids and CBD showed a maximized pattern at over 0.1 mM GABA treatment (Figure 2A). These findings suggest that the impact of GABA on cannabinoid biosynthesis in hemp is concentration-dependent, with initial inhibitory effects potentially leading to increased cannabinoid levels over time.

Significant increases in total THC and CBG were observed on day three in the 1 μM ABA treatment group but not in a concentration-dependent manner (Figure 2B). However, by week four, the cannabinoid levels of all cannabinoid classes were somewhat increased but had no significance across all treatments, indicating that the initial effects of ABA were not sustained over time (Figure 2B).

On day three of SA treatment, the analyses showed similar decrease patterns in all cannabinoid classes in 0.5 mM SA, compared to the control (Figure 2C). In addition, by week four, levels of all cannabinoid classes across all SA-treated groups were comparable to those in the control (Figure 2C). These results demonstrate that the initial SA hormone treatment affected cannabinoid content levels but not cannabinoid accumulation by 4 weeks post-treatment.

### 2.3. Correlation Analysis of Cannabinoid Biosynthetic Genes and Cannabinoid Contents

Next, we explored the relationship between gene transcript profiles (at 6, 12, 18, 24, 48, and 72 h after hormone treatment) and cannabinoid content levels (at 3 days and 4 weeks) in hemp female inflorescences to assess potential links between gene expression patterns and metabolite production. A Pearson’s correlation matrix was constructed to display the correlations between gene transcript levels and cannabinoid content levels.

**Figure 3:**
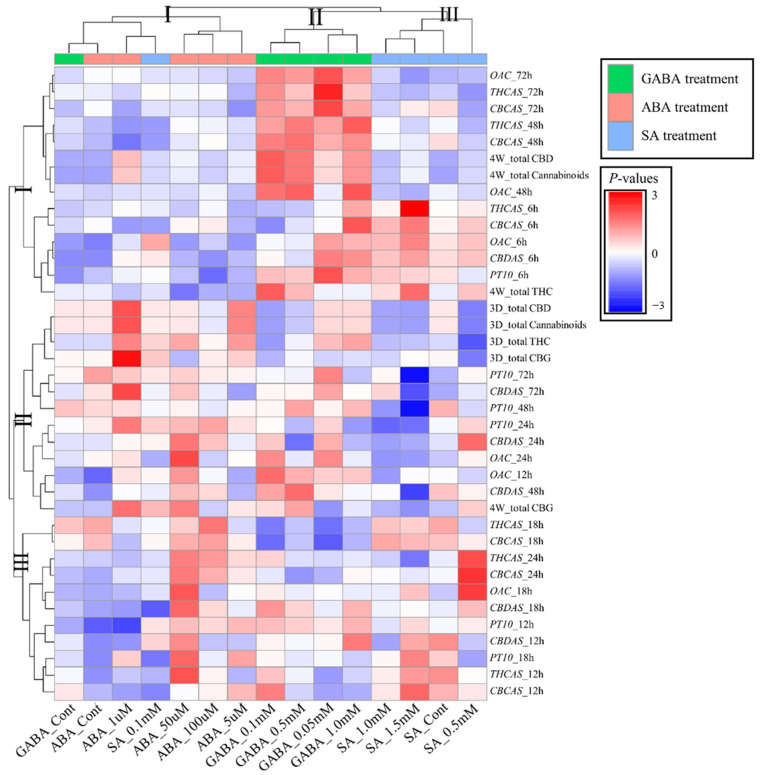
Graphic representation of correlation matrices showing correlations between cannabinoid biosynthetic gene transcript levels and concentrations of metabolites. Red indicates a positive correlation and blue indicates a negative correlation. The size and color of the circle represent strength of correlation. *OAC*, encodes olivetolic acid cyclase; *PT10*, prenyltransferase 10; *THCAS*, tetrahydrocan-nabinolic acid synthase; *CBDAS*, cannabidiolic acid synthase; *CBGAS*, cannabigerolic acid synthase.

Clustering of the treatments revealed three distinct clusters. Cluster I comprisd all concentrations of ABA treatments, indicating a negative correlation in gene expression at 6, 48, and 72 h, and week four cannabinoid levels across these concentrations, which suggests a dose-dependent effect where incremental increases in ABA concentration did not significantly alter the outcome of total cannabinoid at four weeks (Figure 3). However, all cannabinoid classes three days after ABA treatment strongly correlated with 1 µM ABA and *PT10*, *CBDAS*, and *OAC* expression.

Cluster II included all GABA treatments (0.05 mM, 0.1 mM, 0.5 mM, and 1.0 mM), excluding the control. In these concentrations of GABA, strong correlations between gene expression and cannabinoid content were detected in *OAC*, *THCAS*, and *CBCAS* at 72 h post-treatment, and in week four total cannabinoids and CBD.

In contrast, cluster III consisted of all SA concentrations, except for SA 0.1 mM, and broadly showed a negative correlation (Figure 3). Positive correlations were detected in the initial SA response (6 h after treatment) of the five major genes and in the expression of *OAC*, *THCAS*, and *OAC* (18 h and 24 h after treatment), but they did not extend to influencing cannabinoid content levels on day three and week four. Interestingly, the correlation matrix analysis showed that the GABA and SA response at the early expression (6 h) of the five genes displayed similar positive patterns, but an opposite pattern was observed in cannabinoid accumulation at week four.

## 3. Discussion

We examined how treatments with three hormones (GABA, ABA, and SA) at a range of concentrations affected the transcript profiles of five key genes (*OAC*, *PT10*, *THCAS*, *CBDAS*, and *CBCAS*) involved in the cannabinoid biosynthesis pathway in hemp inflorescences. Our results reveal GABA’s fine dose-dependent effects on the expression of cannabinoid biosynthesis genes. In addition, the hormonal effects on the transcript levels of the GABA-mediated sample extended to the accumulation of total cannabinoids at 4 weeks after treatment. These findings were confirmed by a Pearson’s correlation matrix assay. The ABA and SA treatments also altered the transcript levels of the five key genes, whereas their impact on the final accumulation of cannabinoid content was limited. Overall, in this study, GABA was the most effective hormone for gene expression and cannabinoid accumulation in the final harvest.

GABA, as a signaling molecule but not as a phytohormone, plays a preventative role against frost damage in the harvesting stage by stimulating ethylene biosynthesis [29], which enhances plant stress tolerance by improving photosynthesis and activating antioxidant enzymes [30]. The most effective GABA response was observed as an increased accumulation of total cannabinoids at week four post-treatment (Figure 2A). This means that exogenous GABA improved overall plant vigor during flower development [31]. In addition, the strong effects of GABA on *OAC*, *THCAS*, and *CBCAS* expression appeared after treatment with GABA at 0.05 mM and 1.0 mM, exhibiting high expression levels at 72 and 48 h, respectively, in contrast to earlier time points (6–24 h) (Figure 1). These findings suggest that the application of 1.0 mM GABA at 48 h may represent an important induction point, indicating the potential significance of both the concentration and timing of GABA-mediated induction in the regulation of the cannabinoid biosynthetic pathway.

However, cannabinoid accumulations were not much different from the control at day three (Figure 2A). An earlier study by Jalali et al. [32] examined the transcript levels of *THCAS*, *CBDAS*, *OAC*, and *PT* following treatment with 0.1 mM GABA and found the highest transcript levels of the four genes after 72 h post-treatment, which is similar to our results, although the authors used a drug type cannabis called Saghez. However, the positive correlation between *THCAS* expression and THC content that Jalali et al. [32] observed at 3 days does not match our results, in which only a low positive correlation was detected in hemp inflorescence following treatment with 0.05 mM GABA (Figure 2A and 3). These findings indicate that the GABA response in hemp and marijuana can enhance gene expression involved in the cannabinoid biosynthetic pathway and cannabinoid accumulation, but does not alter the intrinsic properties of hemp and marijuana, such as the THCAS and CBDAS enzyme activity that confer discrimination between hemp and marijuana.

Another study reported that weekly exogenous GABA treatment for 8 weeks results in significant changes in the decreased expression of the *OAC*, *CBDAS*, *THCAS*, and *PT4* genes, as well as cannabinoid content levels [33]. Overall, excessive GABA treatment negatively affects the induction of cannabinoid biosynthetic gene expression and cannabinoid accumulation. Hence, a single application of GABA at 1.0 mM concentration during female flower development is the most suitable approach for increasing cannabinoid content levels.

We found that ABA treatment induced the expression of all five studied genes, with varying patterns observed. The *OAC* and *PT10* transcript levels were increased in the 50 µM (18 h) ABA treatments, but *THCAS* expression was decreased in all ABA treatments compared to the control (0 h). The peak transcript level of *CBDAS* was at 72 h after treatment with 1 µM ABA. The expression pattern of *PT10* induced by ABA at 12–18 h is supported by Sands et al.’s study [34], in which ABA treatment activated the promoters of *CsPT1* and *CsPT4*. In the present study, gene expression mediated by ABA treatment had an effect on cannabinoid content levels. Treatment with 1 µM ABA led to the highest concentrations of total THC, CBD, CBG, and cannabinoids at three days, indicating an early stimulatory effect of ABA on cannabinoid biosynthesis, whereas treatment with a greater 5 µM ABA was seen to gradually decrease cannabinoid levels (Figure 2). After 4 weeks, ABA treatment resulted in no significant change in cannabinoid accumulation (Figure 2). A previous study [35] based on an approximately 3 and 30 µM ABA treatment found increased THC content levels in hemp flowers compared to the control after 4 days with three occasional treatments of ABA, but THC content levels following treatment with 30 µM ABA were slightly lower when compared with the 3 µM ABA treatment in the flowers. These results suggest that a high concentration of ABA negatively affects cannabinoid content in hemp.

Another study reported that THC and CBD content levels decreased after 4 days following three doses of 1 and 10 µM ABA treatment in drug-type cannabis plants [36]. It is known that drought triggers the ABA signaling pathway, and drought stress during 2 weeks of flowering led to a decrease in total CBD and THC content levels [36]. Overall, ABA treatment appears to function through different signaling responses between hemp and drug-type cannabis (marijuana), and does not significantly affect cannabinoid accumulation beyond the natural cannabinoid increase associated with the development of female flowers. However, it influences the initial increase in cannabinoid content.

The SA treatments also affected the gene expression of *OAC*, *PT10*, *CBDAS*, and *CBCAS*. In particular, the transcript levels of *PT10* were higher in the 1.5 mM SA treatment at 18 h than in the treatments with SA at lower concentrations. Conversely, treatment with SA at a low concentration (0.5 mM) resulted in the highest transcript levels of *CBDAS* and *CBCAS*. Consistent with this result of *PT10*, a previous study reported that promoters of *CsPT1* and *CsPT4* can be activated by SA [34]. However, negative effects on the expression of *CBDAS*, *OAC*, and *PT* in response to SA treatment have been documented in the flowers of drug-type cannabis [32], indicating that cannabinoid biosynthesis genes respond differently to SA treatments.

According to the differential expression patterns of SA (Figure 1), cannabinoid content levels at 3 days and 4 weeks were similar to or less than in the control (Figure 2C). Garrido et al. [33] found that SA treatment (total eight applications, per week) during the generative stage did not alter the expression of genes involved in the final steps of the cannabinoid biosynthetic pathway, namely *CsOAC-1*, *CsOAC-2*, *CsPT4*, *CsCBDAS*, and *CsTHCAS*, in inflorescences of drug-type cannabis cultivar at 2 weeks before harvest. Additionally, the total cannabinoid in flowers was decreased at 0.1 mM and 10 mM SA treatments. These findings illustrate that the effects of hormone treatments can differ depending on when the treatments are applied, and when samples are collected. Despite the changes in the expression of cannabinoid biosynthesis genes, previous studies and our research did not demonstrate an increase in cannabinoid accumulation. This supports the findings of Park et al. [37], who found no impact on cannabinoid production in response to 5 days of mechanical wounding during the first week of female flowering, induced by the SA hormone. Furthermore, Flores-Sanchez et al. [38] found no significant changes in cannabinoid content levels in cannabis suspension cells treated with SA. Therefore, it can be concluded that SA is not suitable for enhancing cannabinoids in hemp or marijuana at the flowering stage.

Overall, the three hormone treatments (GABA, ABA, and SA) influenced the expression of cannabinoid biosynthesis genes during the early response phase. However, this did not always necessarily lead to an increase in cannabinoid accumulation and synthase activity. The relationship between cannabinoid biosynthesis and accumulation ultimately depends on how energy derived from photosynthesis is utilized. Since GABA enhances photosynthetic efficiency and overall plant vigor, it likely had the greatest impact on cannabinoid accumulation during flower development. In contrast, although ABA and SA temporarily upregulated gene expression, these effects might be offset by the expression patterns of cannabinoid biosynthesis genes during flower development. As a result, they failed to alter the energy-producing capacity of the plant and did not contribute to increased cannabinoid accumulation at harvest. Additionally, ABA and SA may have redirected energy toward the production of other secondary metabolites involved in stress resistance rather than cannabinoid biosynthesis. Therefore, to enhance cannabinoid accumulation in the cannabis plant, it is crucial to maintain a consistent energy supply through photosynthesis during flower development.

Enhancing cannabinoid content is the primary goal in cannabis cultivation and breeding. To achieve this, various exogenous hormonal treatments have been utilized in cannabis plants. Our data and a previous study [28] showed that total THC, CBD, and CBG content increased significantly as flowers matured, reaching peak concentrations at 6–8 weeks post-anthesis and indicating that cannabinoids gradually accumulate during flower development. In this aspect, it is crucial to determine whether a hormone treatment can lead to alterations in related gene expression and ultimately boost cannabinoid content. In this study, we examined the expression profiles and cannabinoid content in female flowers under GABA, ABA, and SA treatment at different concentrations and time courses, and their correlations. Of the treatments tested, single treatment of GABA at 1.0 mM was the most effective in enhancing cannabinoid accumulation at the last flowering stage. However, further studies are necessary to validate the signal crosstalk between the various exogenous hormones in cannabis flowers. This study provides insights for the cultivation of female cannabis flowers and for understanding the effects of plant hormonal treatment during female flower development.

## 4. Materials and Methods

### 4.1. Plant Materials and Growth Conditions

Hemp was cultivated at the Radiation Breeding Farm of the Korea Atomic Energy Research Institute (KAERI; Jeongeup, Jeollabuk, Republic of Korea) under strict approval guidelines provided by the Ministry of Food and Drug Safety (permit number 247). Seeds of feminized hemp ‘Spectrum 303’ were received from Jeonbuk National University and allowed to germinate and grow for 4 weeks, and the seedlings were then transferred into pots (12 cm × 10 cm × 12 cm, top × height × bottom diameter) filled with horticultural soil (Baroker, Seoul Bio, Seoul, Republic of Korea). From this mother plant, numerous individuals were propagated by the cuttings, which were grown for 4 weeks under vegetative growth conditions and followed reproductive growth.

The plants were grown in a smart farm under the following conditions during the vegetative growth stage: air temperature 25 °C ± 2 °C (daily mean temperature), relative humidity ranging from 30% to 50%, 130–150 photosynthetic photon flux density (PPFD, µmol·m^−2^·s^−1^), and a 16-h/8-h (day/night) photoperiod. During the reproductive growth stage (flowering stage), the temperature and humidity remained the same, but the PPFD was adjusted to 350–370 µmol·m^−2^·s^−1^ and the photoperiod was adjusted to 12-h/12-h (day/night).

### 4.2. Plant Hormone Treatments

Plants fully grown under vegetative growth conditions were all placed under the short day. The randomly selected three plants with a similar size were then used to foliar spray with different hormones during each treatment. These three plants were a group of one replicate. The hormone treatments (single frequency, a drenching foliar spray of 25 mL per individual) were applied when the first female flowers appeared (after a reproductive growth period of 2 weeks under a 12 h/12 h photoperiod). The hormone treatments were conducted as described in Jalali et al. [32] and Mansouri et al. [35] with the following modifications: the plants were foliar sprayed with five concentrations of GABA (0, 0.05, 0.1, 0.5, and 1.0 mM), five concentrations of ABA (0, 1, 5, 50, and 100 uM), or five concentrations of SA (0, 0.1, 0.5, 1.0, and 1.5 mM). These three hormone treatments and all experiments were conducted sequentially for each hormone. After hormone treatment, inflorescences were harvested and mixed from the top, mid and bottom of the three plants for one replicate, and either dried to analyze cannabinoids or immediately frozen in liquid nitrogen and stored at −80 °C until total RNA was extracted.

### 4.3. RNA Extraction and cDNA Synthesis

Female hemp inflorescences were harvested seven times (at 0, 6, 12, 18, 24, 48, and 72 h) in sufficient quantities for each treatment, and three biological replicates were used. The inflorescences were immediately frozen and ground to a fine powder using liquid nitrogen and a mortar and pestle. Total RNA was extracted from the ground material using TRIzol reagent (Invitro-gen, Carlsbad, CA, USA). The RNA quantity and quality were determined using a NanoDrop ND-1000 spectrophotometer (Thermo Fisher Scientific, Waltham, MA, USA) prior to DNase digestion. For each sample, 15 µg total RNA was digested in a volume of 20 µL using the Invitrogen DNA-free Kit (Life Technologies, Grand Island, NY, USA) to remove genomic DNA contamination following the manufacturer’s instructions. After DNase I digestion, the RNA concentration was determined using a NanoDrop ND-1000 spectrophotometer. First-strand cDNA synthesis was performed using 1 µg DNase-treated total RNA in a 20 µL reaction using the SuperScript III First-Strand Synthesis SuperMix kit (Invitrogen, Carlsbad, CA, USA) following the manufacturer’s instructions.

### 4.4. Quantitative Real-Time PCR and Heatmap

Gene transcript levels were analyzed using quantitative real-time PCR, which was completed using the CFX96 Real-time PCR system (Bio-Rad, Hercules, CA, USA) and iTaq Universal SYBR Green Supermix (Bio-Rad). Each 20 µL reaction mixture contained primers (10 pmol) and 3 µL prepared cDNA as the template. The thermal cycling program used for amplification was as follows: 10 min at 95 °C; 40 cycles of 15 s at 95 °C, 15 s at 50 °C, and 30 s at 72 °C followed by one cycle of melting. Relative gene transcript levels were calculated using the 2^−ΔΔct^ method [39], with TUB serving as the internal reference gene [40]. The gene-specific primers used for qRT-PCR analyses are listed in Supplementary Table S2.

To generate a heatmap box for each gene and hormone treatment (with time courses), each value was divided by the largest value of 2^−ΔΔCt^ from the whole set of a gene, then averaged with three biological replicates. These average values were multiplied by 2.5, for the range between 0 and 2.5 with a color scale. The largest value is close to red and the smallest value is close to green.

### 4.5. Total Cannabinoid Extraction and HPLC Analysis

For the analyses of cannabinoid concentrations, the inflorescences of three biological replicates were sampled at 3 days and 4 weeks after the hormone treatments (Supplementary Figure S2). The samples were dried at 55 °C for 24 h in the dark, vacuum-sealed, and stored in a drying room at 10 °C until analysis. Dry sample material weighing 200 mg was extracted with 15 mL methanol for 15 min at room temperature. Then, 100 µL of the extract was transferred to a new vial and mixed with 3 mL acetonitrile. Finally, 10 µL of the mixture was injected into the HPLC system.

The HPLC analysis to detect cannabinoids was conducted using a model cannabis HPLC analyzer (CT Instruments Ltd., Calgary, AB, Canada) equipped with a UV/VIS detector (CT Instruments Ltd.). For separation, an SS C18 column (150 × 4.6 mm, 5 µm particle size) (CT Instruments Ltd.) was maintained at a constant temperature of 30 °C by an external thermostat. The mobile phase consisted of a mixture of formate buffer (CT Instruments Ltd., CTIC-0101) and acetonitrile (Sigma-Aldrich, Buchs, Switzerland), diluted at a ratio of 1:4 (*v*/*v*). The separation of cannabinoids was based on isocratic elution, and the solvent flow rate was 1.2 mL/min. The identification of chromatographic peaks was performed by matching their retention times to those of reference standard compounds, and on the basis of absorption at 220 nm. Weight percentage (% *w*/*w*) and milligrams per gram (mg g^−1^) values were automatically calculated using CT Instruments’ software (CTI Log V.1.60.54, CT Instruments Ltd.) and peak height [41].

### 4.6. Statistical Analysis

Each experiment was performed in triplicate and all data are presented as the mean ± standard deviation (SD). The data of chemical analysis and gene expression were subjected to analysis of variance using a Duncan’s multiple range test, and Pearson’s correlation analyses were conducted using SPSS version 20 (IBM Corp., Armonk, NY, USA). For gene expression, statistical significance in a heatmap of Figure 1 denoted with small letters is listed in Supplementary Table S3.

## 5. Conclusions

We investigated how treatments with three hormones (GABA, ABA, and SA) at a range of concentrations affected the cannabinoid content levels of female hemp inflorescences. Quantitative RT-PCR analyses were conducted to determine how the hormone treatments affected the transcript levels of five key genes in the cannabinoid biosynthesis pathway. The results confirm a dose-dependent relationship between GABA and the induction of genes in the cannabinoid pathway, with a critical induction point at 48 h after treatment with 1.0 mM GABA. HPLC analysis revealed that of the three hormones, GABA treatment at concentrations above 0.1 mM led to the greatest increase in total CBD and cannabinoid content levels compared to the control at 4 weeks post-treatment. Additionally, GABA treatment significantly induced the expression of key genes involved in the cannabinoid biosynthesis pathway, indicating that GABA has a distinct and influential effect on both the biosynthesis process and the accumulation of cannabinoid end products.

## Data Availability

The original contributions presented in this study are included in the article/Supplementary Materials. Further inquiries can be directed to the corresponding author.

## Supplementary Materials

The following supporting information can be downloaded at: https://www.mdpi.com/article/10.3390/ijms26073445/s1.
