# Supplementary material for: Effect of Hormonal Treatments on Cannabinoid Content Levels in Female Hemp (Cannabis sativa L.) Inflorescences
Source: Int J Mol Sci. 2025 Apr 7;26(7):3445. doi: 10.3390/ijms26073445 (PMC11989512; doi:10.3390/ijms26073445)
Supplement: Supplementary file 1 [file ijms-26-03445-s001.zip › ijms-3556675-supplementary.pdf]

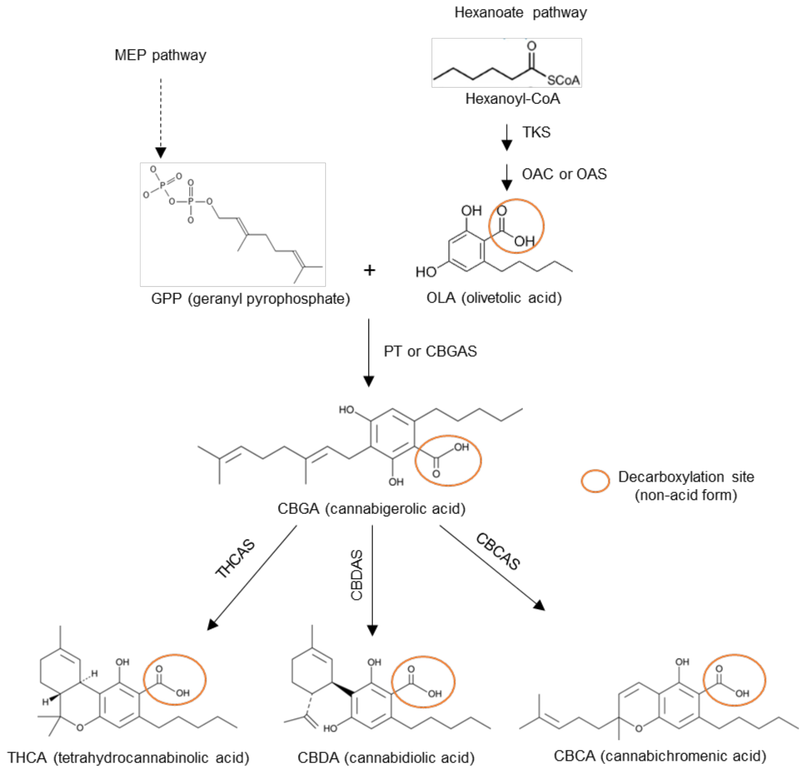

Figure S1. Schematic cannabinoid biosynthesis pathway in *Cannabis sativa* L. Non-acid form of cannabinoids can be generated by heat decarboxylation. 2-C-methyl-D-erythritol 4- phosphate, MEP; TKS, tetraketide synthase (polyketide synthase); OAC or OAS, olivetolic acid cyclase or synthase; PT & CBDAS, prenyltransferase & cannabigerolic acid synthase; THCAS, tetrahydrocannabinolic acid synthase; CBDAS, cannabidiolic acid synthase; CBCAS, cannabichromenic acid synthase.

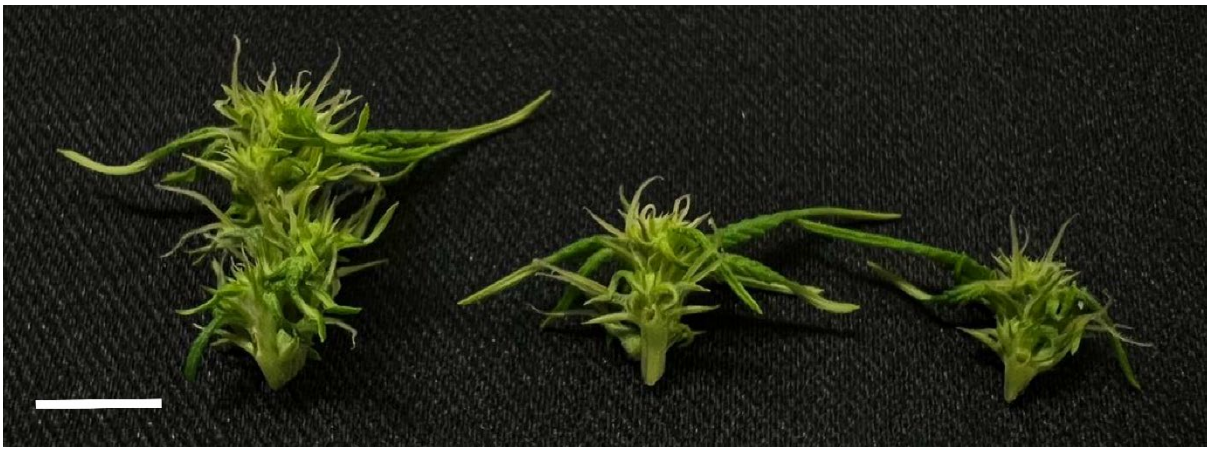

Figure S2. Developmental stage of female hemp inflorescences analyzed in this study (after 4 weeks of hormonal treatment). Inflorescences from the left denote harvest product from top, mid, and bottom, respectively. Bar = 1 cm.

Table S1. Variation of cannabinoid content (mg/g dry weight) in female hemp inflorescences at 3 days and 4 weeks after hormone treatment.

Number	Lines	Results (mg/g dry weight) Total THC	Total CBD	Total CBG	Total Cannabinoids
1	G3D0-1F	2.18	35.84	0.57	44.18
2	G3D0-2F	2.11	37.09	0.66	44.76
3	G3D0-3F	1.76	35.27	0.64	42.29
4	G3D0.05-1F	2.24	49.49	0.65	58.82
5	G3D0.05-2F	2.4	48.93	0.58	58.34
6	G3D0.05-3F	2.41	49.64	0.58	59.15
7	G3D0.1-1F	1.72	35.48	0.59	42.54
8	G3D0.1-2F	1.88	35.31	0.55	42.44
9	G3D0.1-3F	1.41	32.35	0.56	38.65
10	G3D0.5-1F	2.02	40.32	0.65	48.27
11	G3D0.5-2F	2.05	39.49	0.65	47.49
12	G3D0.5-3F	2.03	37.08	0.64	44.6
13	G3D1.0-1F	2.82	53.06	0.62	63.62
14	G3D1.0-2F	2.24	46.64	0.61	55.62
15	G3D1.0-3F	2.29	47.64	0.62	56.84
16	G4W0-1F	2.87	76.77	0.62	92.49
17	G4W0-2F	4.21	83.64	0.73	101.85
18	G4W0-3F	3.46	67.39	0.58	82.04
19	G4W0.05-1F	2.61	71.05	0.53	85.54
20	G4W0.05-2F	2.29	64.62	0.59	77.68
21	G4W0.05-3F	4.02	74.62	0.61	91.53
22	G4W0.1-1F	4.19	84	0.62	102.08
23	G4W0.1-2F	4.08	80.92	0.65	98.41
24	G4W0.1-3F	2.66	76	0.63	91.17
25	G4W0.5-1F	2.96	77.56	0.66	93.65
26	G4W0.5-2F	2.87	77.94	0.62	93.82
27	G4W0.5-3F	4.02	78.24	0.69	95.45
28	G4W1.0-1F	2.56	72.84	0.61	88.46
29	G4W1.0-2F	2.59	73.83	0.62	89.54
30	G4W1.0-3F	3.76	79.63	0.6	97.07
31	A3D0-1F	2.38	61.08	0.72	71.99
32	A3D0-2F	2.44	61.84	0.7	72.92
33	A3D0-3F	1.31	50.39	0.63	58.7
34	A3D1-1F	2.48	63.63	0.92	75.11
35	A3D1-2F	2.6	63.37	0.97	75.06
36	A3D1-3F	2.68	66.55	0.94	78.43
37	A3D5-1F	2.01	48.42	0.64	56.91
38	A3D5-2F	3	76.57	0.87	90.06
39	A3D5-3F	2.46	51.37	0.62	61.04
40	A3D50-1F	2.36	49.08	0.58	58.43
41	A3D50-2F	2.48	46.31	0.58	55.54
42	A3D50-3F	2.45	47.7	0.56	56.54
43	A3D100-1F	1.93	38.16	0.76	45.72
44	A3D100-2F	2.49	51.22	0.75	60.91
45	A3D100-3F	1.95	37.53	0.52	44.95
46	A4W0-1F	2.96	57.11	0.55	68.66
47	A4W0-2F	2.67	50.35	0.57	60.57
48	A4W0-3F	2.13	39.55	0.67	47.86
49	A4W1-1F	3.3	76.12	0.65	90.69
50	A4W1-2F	2.67	72.59	0.67	87.52
51	A4W1-3F	2.42	67.25	0.72	81.11
52	A4W5-1F	3.2	64.23	0.55	78.15
53	A4W5-2F	2.97	58.79	0.64	70.66
54	A4W5-3F	1.96	73.39	0.7	89.71
55	A4W50-1F	2.25	61.74	0.66	74.46
56	A4W50-2F	2.56	67.09	0.66	80.85
57	A4W50-3F	2.68	68.23	0.7	82.52

58	A4W100-1F	3.65	70.39	0.65	85.85
59	A4W100-2F	1.87	44	0.51	53.69
60	A4W100-3F	2.65	73.76	0.64	88.69
61	S3D0-1F	1.69	51.38	0.72	60.6
62	S3D0-2F	1.35	49.72	0.64	58.19
63	S3D0-3F	2.11	45.41	0.71	54.21
64	S3D0.1-1F	2.38	50.49	0.74	60.01
65	S3D0.1-2F	2.17	44.19	0.62	52.74
66	S3D0.1-3F	2.25	47.55	0.8	56.15
67	S3D0.5-1F	1.08	31.58	0.43	38.03
68	S3D0.5-2F	1.49	25.84	0.5	31.24
69	S3D0.5-3F	1.64	35.24	0.57	41.88
70	S3D1.0-1F	2.05	39.8	0.6	47.65
71	S3D1.0-2F	1.84	36.39	0.67	43.65
72	S3D1.0-3F	1.58	28.06	0.54	33.95
73	S3D1.5-1F	1.61	30.79	0.53	37.13
74	S3D1.5-2F	1.92	36.79	0.64	44.18
75	S3D1.5-3F	1.98	35.36	0.8	42.83
76	S4W0-1F	2.89	56.31	0.53	67.7
77	S4W0-2F	2.56	54.09	0.59	64.88
78	S4W0-3F	2.8	52.94	0.53	63.76
79	S4W0.1-1F	3.42	65.06	0.63	79.5
80	S4W0.1-2F	2.43	64.53	0.53	78.68
81	S4W0.1-3F	2.89	61.5	0.79	73.85
82	S4W0.5-1F	3.75	67.76	0.61	82.86
83	S4W0.5-2F	3.24	65.69	0.72	80.07
84	S4W0.5-3F	2.74	57.14	0.59	69.71
85	S4W1.0-1F	3.37	67.15	0.61	81.7
86	S4W1.0-2F	3.13	57.56	0.62	70.84
87	S4W1.0-3F	2.96	60.76	0.54	73.97
88	S4W1.5-1F	3.61	62.36	0.58	76.48
89	S4W1.5-2F	3.57	69.72	0.58	84.94
90	S4W1.5-3F	3.59	63.9	0.56	78.77

G, GABA hormone; A, ABA hormone; S, salicylic acid hormone; 3D, at 3 days; 4W, at 4 weeks; the number before -, hormone concentration each; the number after -, replicates of flowers.

Table S2. Primer pairs used for quantitative real-time PCR analysis of genes related to cannabinoid biosynthesis.

Name	Description	Forward primer (5' to 3')	Reverse primer (5' to 3')
OAC	Olivetolic acid cyclase	CACAGAAGCCCAAAAGGAAG	CAACATGGGCAGGATGAATA
PT10	Prenyltransferase 10	CTGACAACGACCAACATTGC	CAAGAAGGAGCGTACGAAGG
THCAS	Tetrahydrocannabinolic acid synthase	GATCAGCTGGGAAGAAGACG	ATACCACCGTAAGGGTACAACA
CBDAS	Cannabidiolic acid synthase	AGGTGGACACTTTGGTGGAG	TGATTCCGAAGCTTTCTGCT
CBCAS	Cannabichromenic acid synthase	ATGATGCTGAGGGTTTGTCC	TTTCGCATCAATGCTCCATA
TUB	Beta-tubulin	CTCGGCTGAGAAAGCATACC	CCATGCCTAGGGTCACACTT

Table S3. Summarized statistical significance in a heatmap used in Figure 1.

Genes	Times<br>Hormone Conc.	6h	12h	18h	24h	48h	72h
OAC (Olivetolic acid cyclase)	GABA 0.05mM	*a*	*a*	*a*	*a*	*a*	*cd*
	GABA 0.10mM	*a*	*a*	*a*	*a*	*b*	*ab*
	GABA 0.50mM	*a*	*a*	*a*	*a*	*c*	*a*
	GABA 1.00mM	*a*	*a*	*a*	*a*	*d*	*a*
PT10	GABA 0.05mM	*c*	*ab*	*ab*	*b*	*ab*	*c*
	GABA 0.10mM	*ab*	*b*	*b*	*b*	*ab*	*ab*

(prenyltransferase, CBGA synthase)	GABA 0.50mM	*ab*	*b*	*ab*	*ab*	*b*	*ab*
	GABA 1.00mM	*ab*	*b*	*b*	*ab*	*b*	*ab*
THCAS (tetrahydrocannabinolic acid synthase)	GABA 0.05mM	*a*	*a*	*a*	*a*	*ab*	*bc*
	GABA 0.10mM	*a*	*a*	*a*	*a*	*ab*	*a*
	GABA 0.50mM	*a*	*a*	*a*	*a*	*ab*	*a*
	GABA 1.00mM	*a*	*a*	*a*	*a*	*c*	*a*
CBDAS (cannabidiolic acid synthase)	GABA 0.05mM	*bcd*	*abcd*	*acbd*	*abcd*	*abcd*	*abcd*
	GABA 0.10mM	*abcd*	*abcd*	*cd*	*abcd*	*abcd*	*abcd*
	GABA 0.50mM	*ab*	*abc*	*abcd*	*a*	*d*	*abcd*
	GABA 1.00mM	*abcd*	*abcd*	*abcd*	*ab*	*abcd*	*abcd*
CBCAS (cannabichromenic acid synthase)	GABA 0.05mM	*a*	*a*	*a*	*a*	*ab*	*d*
	GABA 0.10mM	*a*	*a*	*a*	*a*	*cd*	*ab*
	GABA 0.50mM	*a*	*ab*	*a*	*a*	*d*	*ab*
	GABA 1.00mM	*ab*	*a*	*a*	*a*	*bc*	*ab*
OAC (Olivetolic acid cyclase)	ABA 1µM	*a*	*a*	*a*	*a*	*a*	*a*
	ABA 5µM	*a*	*a*	*a*	*a*	*a*	*a*
	ABA 50µM	*a*	*a*	*b*	*b*	*a*	*a*
	ABA 100µM	*a*	*a*	*a*	*a*	*a*	*a*
PT10 (prenyltransferase, CBGA synthase)	ABA 1µM	*abc*	*abc*	*fgh*	*efg*	*cde*	*efg*
	ABA 5µM	*ab*	*efg*	*hi*	*bcde*	*bcde*	*cde*
	ABA 50µM	*abc*	*efg*	*i*	*cde*	*bcde*	*efg*
	ABA 100µM	*a*	*def*	*efg*	*def*	*abcde*	*cde*
THCAS (tetrahydrocannabinolic acid synthase)	ABA 1µM	*a*	*a*	*a*	*a*	*a*	*a*
	ABA 5µM	*a*	*a*	*a*	*a*	*a*	*a*
	ABA 50µM	*a*	*bc*	*a*	*a*	*a*	*a*
	ABA 100µM	*a*	*a*	*c*	*a*	*a*	*a*
CBDAS (cannabidiolic acid synthase)	ABA 1µM	*a*	*a*	*a*	*a*	*a*	*c*
	ABA 5µM	*a*	*a*	*a*	*a*	*a*	*a*
	ABA 50µM	*a*	*a*	*b*	*a*	*a*	*a*
	ABA 100µM	*a*	*a*	*a*	*a*	*a*	*a*
CBCAS (cannabichromenic acid synthase)	ABA 1µM	*a*	*a*	*a*	*a*	*a*	*a*
	ABA 5µM	*a*	*ab*	*ab*	*a*	*a*	*a*
	ABA 50µM	*a*	*a*	*cd*	*cd*	*a*	*a*
	ABA 100µM	*a*	*a*	*d*	*ab*	*a*	*a*
OAC (Olivetolic acid cyclase)	SA 0.1mM	*ab*	*ab*	*ab*	*a*	*ab*	*ab*
	SA 0.5mM	*abc*	*a*	*cd*	*ab*	*a*	*a*
	SA 1.0mM	*abc*	*a*	*ab*	*a*	*a*	*a*
	SA 1.5mM	*d*	*ab*	*abc*	*a*	*a*	*a*
PT10 (prenyltransferase, CBGA synthase)	SA 0.1mM	*abc*	*defghi*	*efghi*	*efghi*	*cdefgh*	*efghi*
	SA 0.5mM	*abc*	*fghi*	*efghi*	*bcdefg*	*efghi*	*defghi*
	SA 1.0mM	*cdefghi*	*cdefgh*	*jk*	*abcdef*	*abcd*	*defghi*
	SA 1.5mM	*cdefgh*	*hij*	*l*	*abcdef*	*ab*	*a*
THCAS (tetrahydrocannabinolic acid synthase)	SA 0.1mM	*a*	*a*	*a*	*a*	*a*	*a*
	SA 0.5mM	*a*	*a*	*a*	*a*	*a*	*a*
	SA 1.0mM	*a*	*a*	*a*	*a*	*a*	*a*
	SA 1.5mM	*b*	*a*	*a*	*a*	*a*	*a*
CBDAS (cannabidiolic acid synthase)	SA 0.1mM	*abcd*	*abcd*	*abcd*	*abcd*	*abcd*	*abcd*
	SA 0.5mM	*abcde*	*abc*	*e*	*cde*	*abcde*	*abcd*
	SA 1.0mM	*abcde*	*ab*	*abcde*	*ab*	*abcde*	*abcde*
	SA 1.5mM	*de*	*abcde*	*abcde*	*ab*	*ab*	*a*
CBCAS	SA 0.1mM	*a*	*a*	*a*	*a*	*a*	*a*
	SA 0.5mM	*a*	*a*	*ab*	*d*	*a*	*a*

(cannabichromenic	SA 1.0mM	*a*	*a*	*bc*	*a*	*a*	*a*
acid synthase)	SA 1.5mM	*a*	*c*	*abc*	*a*	*a*	*a*

Statistical analysis was performed by Duncan's multiple range test ($p < 0.05$, $n = 3$). Different small letters indicate statistical significance.
